# Upcity: Addressing Urban Problems Through an Integrated System

**DOI:** 10.3390/s24247956

**Published:** 2024-12-13

**Authors:** Andre A. F. Silva, Adao J. S. Porto, Bruno M. C. Belo, Cecilia A. C. Cesar

**Affiliations:** Computer Science Division, Aeronautics Institute of Technology, São José dos Campos 12228-900, Brazil; andre.silva.60933@ga.ita.br (A.A.F.S.); adao.porto.61025@ga.ita.br (A.J.S.P.); bruno.belo.60934@ga.ita.br (B.M.C.B.)

**Keywords:** smart cities, integrated system, urban problems, pothole detection, modeling with UML

## Abstract

Current technologies could potentially solve many of the urban problems in today’s cities. Many cities already possess cameras, drones, thermometers, pollution air gauges, and other sensors. However, most of these have been designated for use in individual domains within City Hall, creating a maze of individual data domains that cannot connect to each other. This jumble of domains and stakeholders prevents collaboration and transparency. Cities need an integrated system in which data and dashboards can be shared by city administrators to better deal with urban problems that involve several sectors and to improve oversight. This paper presents a model of an integrative system to manage classes of problems within one administrative municipal domain. Our model contains the cyber-physical system’s elements: the physical object, the sensors and electronic devices attached to it, a database of collected problems, code running on the devices or remotely, and the human. We tested the model by using it on the recurring problem of potholes in city streets. An AI model for identifying potholes was integrated into applications available to citizens and operators so that they can feed the municipal system with images and the locations of potholes using their cell phone camera. Preliminary results indicate that these sensors can detect potholes with an accuracy of 91% and 99%, depending on the detection equipment used. In addition, the dashboards provide the manager and the citizen with a transparent view of the problems’ progress and support for their correct address.

## 1. Introduction

In contemporary cities’ urgent and complex urban dynamics, citizens face daily challenges that directly impact their quality of life and well-being. There are problems of different natures, such as defective public lighting, trees at risk of falling, noise pollution, water or sewage leaks, insect breeding sites that transmit diseases, stray animals, improper waste disposal, and potholes in the street. Sensing in cities and intelligent treatment have emerged as an approach to solving these problems [1]. However, within the municipal administration, each problem is addressed within its domain, and often, although parts of the problem or the solution are shared, each system is developed independently. One issue is that despite having parts in common, the challenges that are specific to each scenario prevent managers from building an integrated system. For example, a sector could manage problems through aerial sensing via drone, which could be helpful for visually monitoring essential issues in the city. However, the problem of detecting harmful insects cannot be solved by simply using inspection drones that cannot get close to the breeding ground. Thus, the sanitation team would naturally deal with the insect problem independently.

The evolution of IoT systems indicates that they confine a large volume of collected data to its own domain. Some research has attempted to deal with these large volumes using information fusion, but more than the information itself, the systems are complex, and in practice, each one tends to remain in its known domain [2]. At the current stage of development of Smart Cities (SCs), standards notice that infrastructures common to different domains facilitate the implementation, management, maintenance, and ultimately, collaboration between domains that could benefit from the same methods [3].

Suppose the problem of the replacement of a faulty light bulb. How could the city administration replace a defective light bulb if it does not have the exact location of the bulb? Could citizens report the location of the problem? The first step in dealing with city problems is to identify them correctly. Several problems are not adequately addressed in urban centers due to the lack of a problem identification system, communication system limitations, and solution planning flaws.

In this work, we create an environment for monitoring urban problems by structuring a high-level environment that counts on the city administration’s sensors but can also accept direct citizen interactions. Our proposal does not deal with data fusion, but data fusion strategies would fit in if necessary.

We began developing a use case by addressing the urban problem of potholes in the asphalt of public roads. Potholes put the safety of drivers, cyclists, and pedestrians at risk, causing traffic accidents and damaged vehicles when not properly maintained. The city of São Paulo, a large urban center in Brazil, registered 1086 complaints about potholes in the first quarter of 2024 Proactive public management carries out road maintenance before accidents occur.

Detecting potholes using an automated system is complex. The dimensions and shapes of the potholes vary greatly. Therefore, we developed a machine learning model embedded in a device that can identify potholes by monitoring city streets with aerial sensing via drone or with the contribution of citizens themselves via their cell phones. Just as pothole data are recorded in the system, other problems also input data via appropriate sensing.

The main contributions of this work are the following:

- The modeling of a cyber–physical system using IoT to address urban problems;
- An application for citizens to report urban problems;
- Dashboards to access indicators of problems and ongoing solutions to promote management evaluation;
- Applying these structures in a practical case of detecting road potholes.

The system is validated, indicating easy operation with a relatively simple code.

The article is organized as follows. Section 2 contains a literature review. Section 3 details the modeling of the system. Section 4 presents the elements that comprise the solution for the pothole treatment use case. Section 5 presents the results, and Section 6 discusses threats, difficulties, and restrictions that may impact the system’s effectiveness. The conclusion is in Section 7.

## 2. Literature Review

We divide our literature review into three parts, each one addressing issues directly related to our proposal: (1) frameworks for addressing problems in Smart Cities in an integrated manner, (2) citizen cooperation in a city surveillance system, and (3) techniques for treating and identifying potholes. These three parts provide support for our modeling and final project. Each of the parts is detailed below.

### 2.1. Framework for Addressing Problems in Smart Cities in an Integrated Manner

There is a trend towards integrating different domains in Smart Cities. Complex problems require data from different sources and provide data that can be used in different contexts. However, integration is not a simple task. From the perspective of integration, the study by Barletta et al. evaluated 74 Italian Smart City projects, concluding that most projects involve only one domain, and only one project involves six domains among those raised in the article’s taxonomy [4].

Initiatives are still somewhat timid or integrate large areas within the same domain, such as the work of Karn et al. [5]. The authors set up a framework for the integrated treatment of the problem of water waste in cities. They equipped the city with nine different types of sensors integrated into the entire water treatment architecture that, through a dashboard, signal the sensing situation to managers. This work is a good example of the integration of areas, as it addresses a complex and challenging problem but only from the perspective of the team involved in water issues. Although it integrated related areas, this work did not go beyond its own boundaries, which is necessary for an integrated Smart City project. One contribution of the work was to show the importance of structuring complex problems with the reference architecture’s design and the life cycle’s development.

Wirtz and Muller created an integration framework using an ecosystem model with several layers organized within an organizational and operational model [6]. The complete model is quite comprehensive, including organizational and operational models. When confronting our purpose with this work, we decide to focus our efforts on collaboration and operational models since we will not discuss business models as they suggested.

Classic Smart City organizations divide the approach into the dimensions that make up the city—smart people, smart government, smart environment, smart transportation, smart economy, and smart life—and try to create a common link between the dimensions [7]. Other studies, such as that of Neitorotti et al., classified SCs by providing a classification in related application areas, such as natural resources and energy, transportation and mobility, buildings, life, government, and the economy and people [8]. Attaran et al. added to the application areas some concepts such as security, infrastructure, and the knowledge necessary to cover engineering and integration requirements that are not present in the isolated components. They created submodels for each of the high-level models. The modeling of these last three articles was quite broad, but they only reached the most abstract modeling and did not build a prototype capable of validating the modeling [9].

A modeling framework to support an integrated approach was presented by [10]. The authors used data from New York and trained a neural network to address the problem of predicting crimes in the city. They used data sources from different stakeholders, which could provide important KPIs for addressing the issue. Although it integrates different agencies involved in the subject, it does not go beyond the criminal domain. However, they showed that this type of platform can achieve synergy between different sectors collaborating.

A more in-depth analysis of these articles allows us to classify them into three types of treatment for the problem of domain integration:

- Articles that assume a taxonomy of the dimensions involved in the integrated treatment of SC;
- Articles that create modeling on how a system could integrate the constituent elements of the SC;
- Articles that carried out some implementation that seeks integration.

Table 1 summarizes this study.

Our study should be placed in the Modeling column because it addresses both collaboration and operational dimensions. Furthermore, it belongs in the Implementation column since it focuses on urban issues in general, specifically tackling pothole problems.

### 2.2. Citizens’ Engagement

Our study of this topic shows that strengthening initiatives requires citizen engagement. Citizen awareness, responsibility, and commitment are crucial to popularizing the Smart City concept. Even though Smart Cities are well organized and use advanced technologies, social awareness is required [11]. One of the dimensions highlighted in studies of a resilient city is precisely the citizen and cooperation. Success is a two-way street: citizen engagement brings benefits to the designed system, and a good system causes greater citizen engagement as shown in studies of cities in Italy [12], Canada [13], and Saudi Arabia [14]. Innovative experiences have been carried out, such as co-creating digital platforms that aim to go a step further in achieving this engagement, which in practice is not easy. Citizens need to be not just participants but co-governors of the public space [15]. Using applications available to citizens is part of the participatory process that can give them ownership of their city. The following factors can influence this: (1) political actions to encourage participation with inclusion and openness; (2) research actions to discover how to engage and empower citizens [16]; and (3) initiatives for co-creating projects in the planning process [17].

Political actions in large cities are the most relevant, as there is a negative relationship between the density of a municipality’s population and citizen engagement: smaller municipalities show greater citizen engagement [12].

These studies have strengthened our idea of not leaving the identification of problems solely as the responsibility of public management but having citizens as participatory agents in managing these problems to which they are closest. The public administration must clarify to citizens the benefits of their participation and the system’s ease of use. On the other hand, citizens must also invest themselves in the role of co-governor and effectively use the system designed for them.

### 2.3. Techniques for Treatment and Identification of Potholes

The application of AI in dynamic surveillance systems is advancing. In our case, detecting a problem statically and with a drone at low speed is feasible. Nguyen’s work used AI in the drone itself to select the data of interest to send to the cloud [18]. This preprocessing reduces both the stored data and the transmitted data. In our case, running the model locally and transmitting the result to the cloud with just a photo of the pothole and metadata would be possible. Lee D. et al. showed that the model may miss important frames in real-time detection systems, resulting in inaccurate or outdated detections due to the rapid change of the scene and the greater demand for real-time processing. Hardware with greater processing power, such as a more robust GPU, more RAM, or processors optimized for neural network inference, allows the system to process more frames per second (FPSs) and better keep up with the input frame rate, reducing the need to discard frames [19].

Samadzadegan et al. used a UAV system to detect problems on paved roads [20]. One of the types of issues is the pothole. They used YOLOv8 as a platform and achieved an accuracy of 81%. YOLO (You Only Look Once) is a widely used generalized object representation. The Ghost Convolution algorithm was recently incorporated, reducing computational complexity without compromising detection accuracy [21]. Object recognition is a sensitive point in the project, which needs to detect road faults accurately, in a practical and scalable way, even in low-light or high-traffic density scenarios. Before choosing YOLO, we evaluated other algorithms for object detection, such as SSD (Single-Shot MultiBox Detector) and Faster R-CNN. Tests with Faster R-CNN indicate higher accuracy in specific conditions but lower speed in dynamic and real-time scenarios. SSD shows good efficiency, but its accuracy is lower for detecting small or complex objects such as a pothole [22]. In addition to offering a better balance between speed and accuracy, YOLO has demonstrated wide acceptance and support from the community, ensuring a feature-rich ecosystem and constant updates.

Combining data sources with many sensors abroad in the cities’ environment is challenging. Information fusion methods should be applied to aggregate data, enhancing the detection of problems [2]. In our study case, we should combine metadata from the cell phone with metadata from the drone because both can detect the same pothole. The sources can be different, but so can the recognition model. For simplicity, in our case, the app just checks the coordinates of the pothole location to detect that the same pothole was detected by both sources.

Ullah et al. presented an AI of Things (AIoT) assisted two-stream neural network for anomaly detection in surveillance video data, using a BD-LSTM layer to classify the anomalies [23]. For the case of potholes, the integration of spatial and optical flow features employed in the paper could be adapted to improve capture accuracy.

The work of Kim et al. pointed to other methods of pothole detection that the administration could employ, such as a vibration detector or laser scanner, which would cost more [24]. It is an ongoing investigation, where different methods apply for different applications. Periodically, technological innovation should be compared with the project’s status.

Having an overview of the approaches in the areas where our problem intersects, we move on to modeling the system.

## 3. Modeling UpCity: Urban Problem Treatment System

This section addresses the modeling of the system. As a consequence of the modeling, a prototype is built, and Section 4 details its construction.

**Figure 1:**
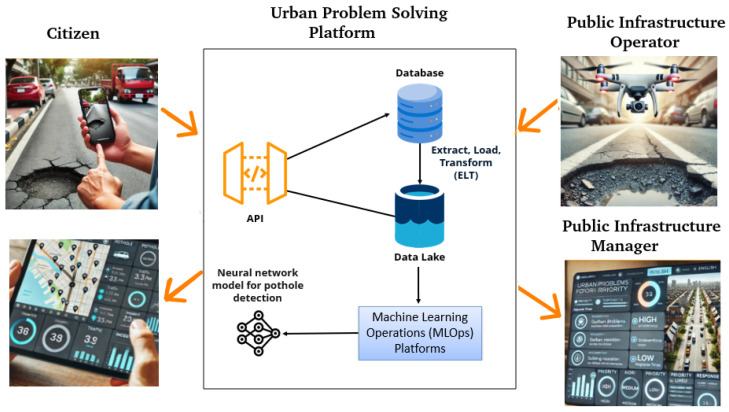
Context diagram of UpCity: urban problem treatment system.

The modeling follows the recommendations of the European IoT-Architecture (IoT-A) project. More than 50 scientists and researchers contributed to developing an “Architectural Reference Model” (ARM) for the Internet of Things [25]. One of the first recommended actions is selecting the relevant physical entity for the specific scenario. In our use case, we define that the pothole is the physical entity. The pothole’s spatial coordinates identify it. Figure 1 provides the context of the project. It is a free-form diagram that shows the external interfaces of the system’s design.

In the center of the figure is UpCity: the code and related databases with the machine learning models. The Public Infrastructure Operator and the citizen help identify the problem given input to the system. The Public Infrastructure Manager and the citizen can access specific dashboards to monitor the progress of solutions. As we started with the pothole problem, we illustrate the pothole, but several problems can enter into the system with local sensing.

This system stores the data in a database designed for easy access. An application has been developed to display these data to citizens and managers, empowering them with the necessary information.

Next, in the modeling, we identify the stakeholders. Table 2 lists the main stakeholders. The influence of the stakeholder on the system is identified as low, medium, and high, which is not indicative of the degree of interest in the project. For example, the city’s senior management is highly interested in this system but has a medium influence on the project’s decisions, which tend to be more technical than political. The degree of influence of each stakeholder is a strategic decision.

**Figure 2:**
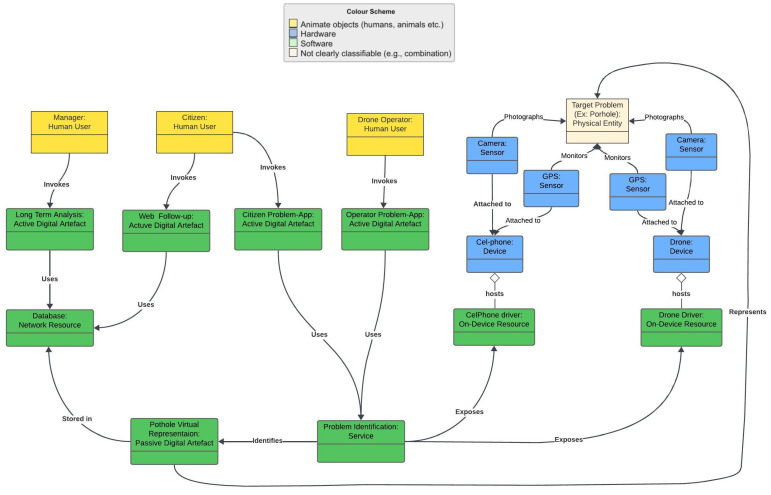
System modeling with UML.

Figure 2 illustrates the system modeling using a UML (Unified Modeling Language) diagram that captures the main elements of the IoT ecosystem to a certain degree of abstraction. This diagram also uses the guidelines of the IoT-A project, capturing the relation between physical elements and cyber elements. The main benefit of this diagram is that it integrates both physical and cyber elements in the same picture using a color representation to differentiate each element. The physical elements use beige, the hardware elements use blue, the human users are in yellow, and the software elements are in green. Elements of the system infrastructure do not appear in this diagram so as not to hinder its understanding. The other elements will appear in the architectural details.

Each of the diagram elements is mapped into a class. The classes are as follows:

- Physical entity: in our case, the pothole (in beige).
- Devices: hardware composed of sensors and controllers (in blue).
- Resources: software components that provide supporting functionality running (1) on-device to capture image localization or (2) remotely as the urban problem database available via a network.
- Passive Digital Artifacts: a digital representation, where the physical pothole has a digital representation in the system corresponding to the images stored and the coordinates of the physical entity (in green).
- Active Digital Artifacts (ADA): running code, i.e., software applications, agents, or services. There are four ADAs in the diagram: (1) citizens app to identify the urban problem; (2) citizens app to follow up with the identified problem; (3) operator app to identify the urban problem via administrative interface; and (4) manager dashboard, including long-term analysis running remotely.
- Human User: three classes of humans interact with the system—the citizen using cellphone apps, the equipment operator, and the administrator using long-term analysis.

The next step in the modeling process is to gather relevant information for each diagram element the system must control. Data processing is an integral part of the project; it is necessary to manage the following:

- The data of the physical objects brought into the cyber world;
- The authenticated users;
- The applications that must be controlled and maintained;
- The databases and the data lake.

For example, from the point of view of the physical object, the hole, the minimum information that the system should control is the following:

- Unique identifier (ID);
- Geographic coordinates (latitude and longitude);
- Date and time of identification;
- Dimensions of the hole (width, length, and depth);
- Current status (repaired and not repaired);
- History of interventions;
- Attached photos and videos.

With the minimum information gathered, we can proceed to the next step and build each part by discussing this structure with the stakeholders.

## 4. Building the System for the Use Case: Identifying Potholes

We use the model presented in the last section as the reference to build the target system. We start with the physical devices: a cellphone and a drone with a GPS sensor and a camera with respective drivers to deal with the information gathered by them.

Then, we proceed using the Standard ISO/IEC/IEEE29148:2018 that identifies information items related to requirements engineering and their content, including characteristics and attributes, in the context of system and software engineering [26]. Table 3 lists the software components developed. They are categorized by the kind of requirement as recommended by the standard. The categories are (1) purely functional and (2) not functional, dealing with security, performance, or scalability issues.

### The Hardest Part of the Use Case: Identifying Potholes

The project’s biggest challenge is developing a neural network model for hole detection. We create the model using advanced machine learning techniques to analyze images containing potholes.

YOLO (You Only Look Once) performs generalized object representation more effectively without precision losses than other object detection models [12]. We consider the possibility of using YOLOv8 instead of YOLOv5 since a more recent version could have higher accuracy in the results. YOLOv8 has a different architecture from its predecessor and, in general, produces better results, but three arguments support our choice for YOLOv5:

1. Some studies using YOLO for mobile and aerial images—our case—have practically a tie between the two. Sary et al. showed that the performance value of the YOLOv8 model is greater than the YOLOv5 model for precision at 2.82% and for F1-score at 0.98%, but for the recall performance value, YOLOv5 is greater than the YOLOv8 model with a difference of 0.54% [27]. A similar result was found in [28].
2. The study by Peserico and Morato showed that if there were no GPU but CPU only—our case—YOLOv5 would take half the time for model inference of YOLOv8 [29].
3. Due to its maturity time, YOLOv5 has a much higher number of already documented and widely available solutions than YOLOv8. The first stable version of YOLOv8 was released in 2023, indicating that it is in the early stages of community adoption.

Although we opt for YOLOv5, the model to be adopted should always be evaluated, and this task should be part of the project life cycle.

The process of training a neural network can be summarized in three steps. It begins with data preprocessing, where data are checked, normalized, and divided into training and testing sets. The second step accomplishes the training process: the model iterates until convergency, achieving the desired accuracy. This step results in the model learning from the data to accomplish the task. Finally, in the third step, the evaluation of the test data checks performance before deployment. Each step is developed in our project, preceded by the environment preparation.

**Figure 3:**
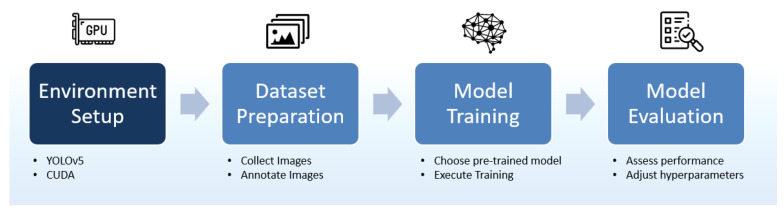
Pothole identification process.

Figure 3 illustrates the steps used in the pothole identification process, whose respective challenges and solutions are described below.

The following is a brief explanation of each step.

**Figure 4:**
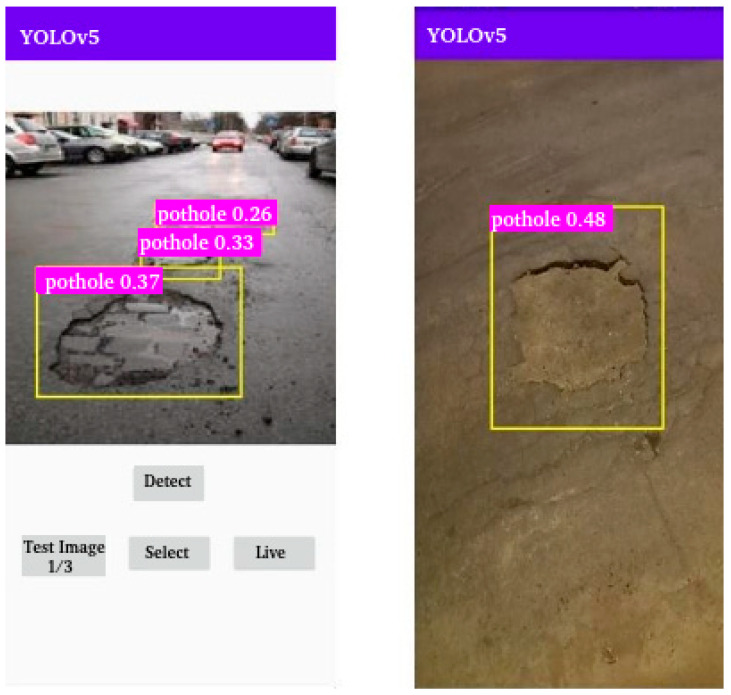
Steps of the pothole identification process.

- Step 1: Environment Preparation:
  We install the necessary tools on Ubuntu 22.04.4: YOLOv5 and CUDA for efficient image processing on an NVIDIA GeForce 3060 8GB video card configured with 3584 CUDA cores.
- Step 2: Dataset Preparation:
  YOLOv5 has pretrained models on a widely known dataset such as COCO (Common Objects in Context) that can identify 80 ordinary object classes, but the class “hole” is not included. Therefore, we have to train the model for this task. We obtain a database from Roboflow with 2395 images of potholes that has already been annotated and preprocessed. This dataset provides a robust and well-structured basis for training. Figure 4 illustrates photos of potholes taken with the cell phone inserted in YOLOv5.
- Step 3: Model Training:
  The training is performed in Python using the parameters shown in Table 4.
  YOLOv5 variants are designed to meet different needs for accuracy and computational performance. The variants with their characteristics range from YOLOv5n, with approximately 1.9 million parameters, ideal for devices with limited resources, such as microcontrollers and IoT devices, to YOLOv5s, YOLOv5m, and YOLOv5L, the largest model with 86.7 million parameters, used in applications that demand maximum accuracy and robust computational resources. Since we intend to use these models on mobile phones and drones, we choose the YOLOv5s version, suitable for embedded systems and mobile devices, and the YOLOv5m version, which provides greater accuracy while maintaining moderate computational requirements [30].
- Step 4: Model Evaluation:
  After training the model, we start to apply it in the tests. Table 5 shows the main results.
  There are two variables shown in the table regarding accuracy:
  – mAP05: a measure of the model’s accuracy considering only the “easy” detections without overlapping objects.
  – mAP05-095: a comprehensive view of the model’s performance across different levels of detection difficulty.
  There are two variables shown in the table regarding errors:
  – Train Obj Loss: Error in object detection during training.
  – Validation Obj Loss: Error in object detection during validation.

Note that the error rates are very low, and the success rates are very high. Given this difference in results, we choose YOLOv5s for our devices because the final results are similar, and it saves time and space.

The mobile application developed for the citizen’s cell phone has among its functionalities the ability to detect the location simply by pointing the cell phone and obtaining a real-time image or by using an image from the cell phone’s photo gallery that also contains the location of the photo.

This application was developed for Android using the CameraX and PyTorch libraries. This link appears twice: here and on the Data Availability Statement. I suggest removing it here.

## 5. Results

We integrated the AI model with the application. With our applications, we ran some simulations of the execution to test the feasibility of applying the model with near-real-time detection running at the edge. We focused on inference time, which measures the time required for the model to process an image, perform object detection, and calculate bounding boxes limiting the object’s area and classifications. This time is essential in measuring the system’s latency and responsiveness. The tests were performed on a cell phone with a 2.0 GHz octa-core processor and 4 GB of RAM. The YOLOv5m model, with 640 × 640 pixel images, had an inference time between 1573 ms and 1667 ms. The YOLOv5s model, being simpler and lighter, presented significantly lower inference times, between 736 ms and 753 ms, resulting in lower latency and suitability for edge applications. The inference time was acceptable for detecting potholes while the citizen is walking, and the application functioned efficiently. However, when used with a moving vehicle, the application had difficulty performing detections, compromising accuracy in dynamic situations.

**Figure 5:**
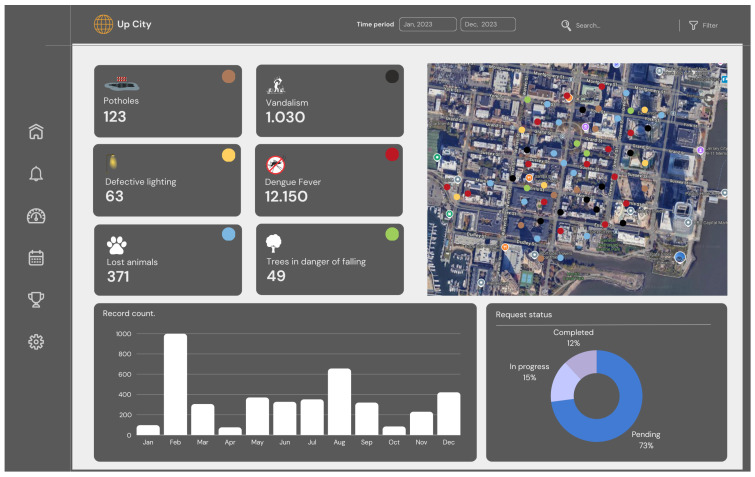
Citizen’s dashboard.

Using simulation data, we built two dashboards to satisfy stakeholder’s requirements. Figure 5 illustrates the citizen dashboard for monitoring all the problems entered into the platform. The indicators present on this screen are as follows:

- Total urban problems by type: potholes, vandalism, defective public lighting, cases of dengue fever, lost animals, etc.
- Interactive map: shows the geographic distribution of problems throughout the city.
- Count of recorded occurrences: shows the number of occurrences per month during the year.
- Request status: shows the proportions of pending, ongoing, and completed requests.

The total number of issues is summarized, but navigating the interface to detail a specific issue is possible. The transparency that must be present on these screens is essential for citizen engagement. The citizen will not contribute to the system if they do not trust these data.

**Figure 6:**
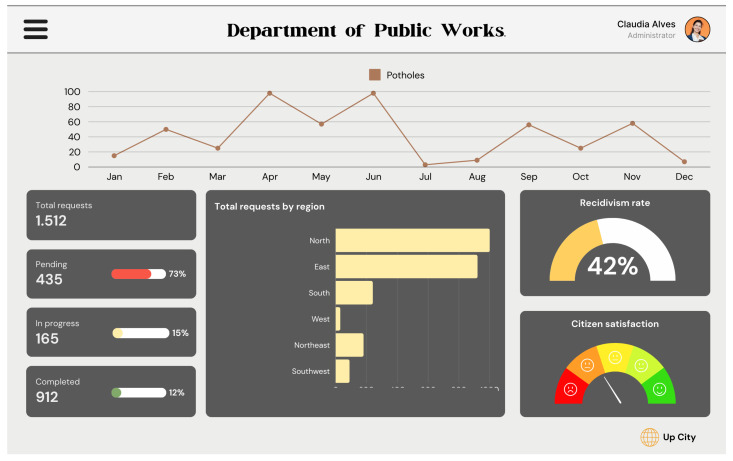
Public infrastructure manager’s dashboard.

Figure 6 illustrates the administrator’s dashboard, which is also used to monitor all issues entered into the platform. However, it has more data than the citizen’s dashboard because it allows for the adequate management of all issues.

The indicators present on its screen are as follows:

- Total number of requests: Total number of requests received.
- The requests status: Total requests organized by status (pending, in progress, and completed).
- A line graph showing the potholes reported over the months.
- Total requests by city region: A bar graph showing requests by region (North, East, etc.).
- Recidivism rate: A rate indicating how often resolved issues reappear.
- Citizen satisfaction: A measure of citizen satisfaction with resolutions.

## 6. Discussion

To ensure a comprehensive understanding of the potential challenges in the development and implementation of the project, we identified threats, difficulties, and restrictions that may impact its effectiveness and adoption by stakeholders. Below are the main points that require attention due to the risks involved:

1. Stakeholder Collaboration
  The system’s efficiency requires the ability of authorities to respond promptly to the reported problems. A quick and effective response strengthens citizens’ trust in the platform and encourages continued use. An integrated problem-reporting system is only helpful if these problems are effectively resolved.
  We must have Smart City building processes where citizens are not mere participants but collaborators in developing and governing these spaces. These processes must be iterative and dynamic, with citizens’ decision-making possibilities. Experiences with Smart City projects that had these objectives show some progress but limited results, which indicates that there is still much to be done in this field [15].
2. Protecting the identity of the citizen
  A classic challenge in the development of public systems is that, on one side, it would be desirable to identify the citizen and hold him responsible for identifying the problem, thus avoiding false users reporting false problems. On the other hand, preserving the citizen’s identity for privacy is desirable. Additionally, the surveillance system uses images that can expose sensitive user information. Privacy is a concern throughout the project.
3. Protecting the system against cyber attacks
  The success of the system requires considering security throughout the development life cycle. The development team must ensure the code is not vulnerable and the access is controlled, conduct penetration testing, and continuously monitor the environment to quickly detect and mitigate attack attempts.
4. Challenges in identifying the problem
  - Identifying problems involves sensors that may fail and trained systems that may issue false positives or negatives that compromise trust in the system. Additionally, other object detection models should be applied in real-world scenarios to compare with our experiments using YOLOv5. The constant adjustment of algorithms and rigorous testing in real scenarios must be carried out.
  - In some cases, the problem should be identified at the edge, without the support of the cloud, and this can be very challenging from the point of view of computational load. Response performance must also be constantly reassessed to avoid overloading the edge or delays that compromise the response.
  - The same proposed modeling could be applied to other urban problems, such as detecting poor street lighting or improper waste disposal. Both drones and citizens can detect these two problems. Another class of problems requires other sensing, such as noise pollution. In this type of problem, the citizen’s contribution is less relevant since they can report it but cannot prove its occurrence. To accommodate this new class of issues, the changes in the modeling and in the system itself would be minimal. However, more research should be dedicated to problems that are not immediately sensing sensitive, such as trees at risk of falling.
5. Operational challenges
  Dynamic environments bring challenges inherent to constant change. Some drone models, for example, cannot fly on rainy days. In this case, only reports from citizens are used. The application hosted on devices (cell phones or drones) may require new drives for different hosting environments. This type of operational issue also requires constant evaluation and maintenance.

## 7. Conclusions

In this work, we created a model at a certain level of abstraction that allows the various stakeholders to interact to identify problems, forward solutions by the competent authorities, monitor the problem by citizens, and monitor the respective managers responsible for the smooth running of the solutions.

After modeling, software prototypes were developed to test the various modules indicated in the modeling. The use case for detecting potholes on municipal highways was fully developed. Tests of an embedded AI model indicated, in the worst case, an accuracy of 91% when there was an inevitable overlap of objects in the scene being detected by the lowest-performing model. When the object was clearly defined, and there were computational resources for detection, the accuracy reached 99%.

Dashboards were developed to collect the generated data and allow citizens and municipal managers to monitor the problem.

Prototyping indicated that by replacing a problem such as potholes with another urban problem, the entire software framework developed could be used, with some specificity addressed in a particular subsection of the application.

Future work would expect to deploy the prototype in a pilot city to test citizen acceptance and effective engagement in the system. Furthermore, after a period of deployment and familiarization, managers could evaluate the dashboards for their effectiveness in responding efficiently to problems detected.

## Figures and Tables

**Table 1:** Different approaches to SC systems integration.

Literature	Taxonomy	Modeling	Implementation
Wirtz et al. [6]	Six dimensions	Modeling with a high-level integration	–
Samarakkody et al. [7]	Six dimensions and subdimensions	–	–
Neitorotti et al. [8]	Six dimensions	–	–
Attaran et al. [9]	Six dimensions and subdimensions	Modeling integrates domains horizontally	–
Westraadt and Calitz [10]	–	Modeling within government dimension	Implementation of Crime Prediction
Karn et al. [5]	–	–	Implementation of Wastewater Treatment
Barletta et al. [4]	Eight dimensions	Modeling integrating dimensions with technologies	–

**Table 2:** Principal stakeholders and their roles.

Stakeholder	Role	Influence
User	identify urban problems and monitor their treatment. The feedback is essential	High
Public Infrastructure Operator	Identify problems and promote repairs	High
Public Infrastructure Manager	Define priorities for responding to open calls	High
Builders	Construct and deploy the system from specifications	Medium
Suppliers	Build and/or supply the hardware, software, or infrastructure on which the IoT system will run	Low
Senior city administration	Monitor the treatment of problems in the city as a whole	Medium

**Table 3:** Software components of the developed system.

Component	Requirement	Description
Citizen App	Functional	Application citizens use to report urban problems. Coupled with a neural network model for pothole detection
Public Issues Dashboard	Functional	Panel with public management indicators containing updated indicators of problems and solutions in progress and completed
Public Works Department System	Functional	System used by the Maintenance Manager to integrate with the project APIs
API Gateway	Not Functional	Proxy that mediates requests that reach the REST resource layer: controls the traffic of requests
JWT - Json Web Token	Not Functional	For data consultation and insertion: guarantees the integrity and confidentiality of information during communication
TLS/SSL with HTTP	Not Functional	Implemented between the application used by citizens, public authorities, and the API Gateway
Web Application Firewall	Not Functional	Protect APIs from vulnerability exploitation
Load Balancer	Not Functional	Distributes traffic across multiple server instances or containers, ensuring high availability, scalability, and performance
Event Driven/Messaging Broker	Not Functional	Asynchronous broker, for the event-driven architecture; allows communication between services in a decoupled and resilient manner
UC-API-Citizen	Functional	A microservice that handles citizen interactions
UC-API-Repairer	Functional	A microservice responsible for functions related to repairers
UC-API-Updater	Functional	A microservice responsible for managing updates in the system. It processes event queues in the event-driven architecture
UC-API-Indicators	Functional	A microservice that provides indicators and reports
Database	Not Functional	Store data entered and updated by citizens and the Department of Public Works
Data Lake	Not Functional	Centralized repository that allows storing data in any format and volume, facilitating data processing and analysis
Neural Network Model for Pothole Detection	Functional	Used by the Citizen Application and the UC-API-Repairer to detect potholes
Machine Learning Operations (MLOps) Platforms	Functional	Responsible for managing the entire life cycle of training the neural network model for pothole detection. The trained models are coupled to the Citizen Application and the UC-API-Repairer

**Table 4:** Model training parameters.

Parameter	Value
Image Resolution	640 × 640
Learning Rate	0.000298
Epochs	100
Batch Size	16
Device	GPU 0

**Table 5:** Model evaluation.

Parameter	YOLOv5m	YOLOv5s
Train Obj Loss	0.0078175	0.00973
Precision	0.98179	0.96114
Recall	0.99841	1.0
mAP05	0.99409	0.99353
mAP05-095	0.94292	0.91121
F1-Score	0.99003	0.98018
Validation Obj Loss	0.003751	0.00453
Model size	42.2 MB	14.1 MB

## Data Availability

Thanks to Roboflow, which offers one of the largest open-source computer vision datasets and APIs. We used data from the following Roboflow Dataset: https://universe.roboflow.com/pothole-mnc0h/pothole_v3-okobj (accessed on 24 November 2024). This application is available at https://github.com/andreferreira60932/app_pothole (accessed on 24 November 2024).

## Abbreviations

The following abbreviations are used in this manuscript:

ADA	Active Digital Artifacts
IoT-A	European IoT-Architecture project
KPI	Key Performance Indicator
SC	Smart Cities
UAV	Unmanned Aircraft Vehicle
UML	Unified Modeling Language
